# Simplified Chinese adaptation of the Yale Physical Activity Survey: reliability and validity measurement in mainland China elderly women

**DOI:** 10.1186/s12889-022-14044-5

**Published:** 2022-08-31

**Authors:** Yuan Yang, Chen Wang, Mingchao Xu, Xiaoke Zhong, Xiaoxia Yuan, Shoufu Yan, Changhao Jiang

**Affiliations:** 1 College of Physical Education and Sports, Beijing Normal University, Beijing, 100875 China; 2 The Center of Neuroscience and Sports, Capital University of Physical Education and Sports, Beijing, 100191 China; 3 School of Kinesiology and Health, Capital University of Physical Education and Sports, Beijing, 100191 China

**Keywords:** Mainland China, Elderly women, Reliability and validity, Physical activity, YPAS

## Abstract

**Objective:**

To verify the reliability and validity of the simplified Chinese version of the Yale Physical Activity Survey (YPAS) scale in the measurement of physical activity of elderly women in mainland China to provide a new standardized scale for evaluating the level of physical activity.

**Methods:**

Twenty-one healthy elderly women completed all the research procedures. The participants completed the questions on the YPAS and wore an Omegawave Sport Technology®System diagnostic system that recorded maximum oxygen uptake and the adaptation index of the energy metabolism system. The reliability of the YPAS was evaluated based on the consistency of the two measurements (pre-test and post-test), and its validity was verified based on the relevant indicators in the Omegawave diagnostic system. Descriptive statistics, intraclass correlation coefficient and Pearson correlation analysis were used. The significance level was set at *p* < 0.05.

**Results:**

In terms of reliability, the Pearson correlation coefficient and ICC of the total physical activity time of YPAS (*r* = 0.917, ICC = 0.897) was high. In terms of total calories, the Pearson correlation coefficient and ICC of the two test results was high (*r* = 0.958, ICC = 0.934). On the YPAS total index, the Pearson correlation coefficient and ICC of the two test was high (*r* = 0.930, ICC = 0.920). In terms of validity, there was a moderate correlation between the energy metabolism system adaptation index and the YPAS-total time (*r* = 0.472) and a moderate correlation with total calories (*r* = 0.472). There was a high correlation between the YPAS-total index and the maximum oxygen uptake (*r* = 0.782).

**Conclusion:**

The simplified Chinese version of the YPAS can measure the level of physical activity and energy metabolism of elderly women in mainland China. It is a reliable tool for measuring the physical activity of the elderly.

## Introduction

The physical activity of individuals decreases with age [1]. Research shows that adequate physical activity is one of the best ways to promote the physical and mental health of elderly individuals and improve their quality of life [2]. Therefore, accurate measurement of the level of physical activity of the elderly is of great significance for evaluating effective intervention strategies designed to improve health and for exploring the dose effects of specific types of activity [3]. However, measurement of physical activity levels in the elderly is currently most often performed using tools designed for young individuals [4]. These measurement tools do not take into account the common types of activities engaged in by the elderly population and therefore do not accurately evaluate the level of physical activity in the elderly. In addition, the relevant measurement tools are not easy to manage and cannot be used to obtain large-scale group measurements [5, 6].

A standardized scale is a feasible method for evaluating physical activity in large-scale populations [7]. Objective evaluation of the physical activity of the elderly using such a scale provides a basis for formulating public health policies that benefit the elderly [8]. The Yale Physical Activity Survey (YPAS) was specifically designed to investigate the physical activity of the elderly. It is one of the classic scales used to measure the physical activity of elderly individuals worldwide. The reliability and validity of the Yale questionnaire have been tested using various methods. Król-Zielińska et al. [9] tested the reliability and validity of the YPAS in a group of 104 Polish elderly aged 65–89 using accelerometers and found that its reliability in measuring total calories consumed, total time spent in physical activity, and leisure walking index was high. In terms of validity, it was found that there was a correlation between the calorie consumption measured by accelerometer and the calorie consumption reported in the YPAS.

In the application of standardized scales, test validity is one of the key indicators. An objective testing instrument is used as the criterion for verification of the physical activity scale. The validity of the verified scale is the key factor in determining whether the scale can be used in investigations and in research [10]. The validity of the YPAS has been verified in groups of elderly individuals living in various countries. For example, Martin et al. [11] studied the validity of the YPAS in 44 Spanish elderly participants based on the 6-minute walking test and found that the YPAS has good validity. Semanik et al. [12] tested 310 American patients with arthritis using the correlation between accelerometer evaluation and the YPAS index. It was found that the YPAS score was highly correlated with the objectively measured level of physical activity, verifying the validity of the YPAS.

In the selection of the criterion used to validate the YPAS, previous research reports mainly used physiological conditions [13] and accelerometers [14] as the criteria for verification. Di Pietro et al. [15] showed that there was a correlation between the related activity index of YPAS and maximum oxygen uptake; Young [16] et al. found that there was a significant correlation between maximal oxygen uptake and the activity index indicated by the YPAS.

To date, the simplified Chinese version of the YPAS has not been studied. The standardized scale used in the YPAS is conducive to comparative analysis with other ethnic groups, allowing investigators to study the factors that determine the level of physical activity of the elderly and to establish a theoretical basis for designing intervention measures that promote a healthy lifestyle. In addition, across most countries, women are less active in physical activity than men. Therefore, we need to accurately grasp women’s participation in physical activity, so as to promote the overall population health [17].

To fill this research gap, this study verified the reliability and validity of the simplified Chinese version of the YPAS in measuring the physical activity of elderly women in mainland China to provide a new tool for evaluating the level of physical activity. This study assumes that the simplified Chinese version of the YPAS has reliability and validity similar to those of the original version and finds that it is a tool that can be used to accurately evaluate the level of physical activity of women in mainland China.

## Methods

### Participants

Twenty-one elderly women residing in a community of Beijing participated in the study. The participants were 60 to 72 years of age (mean age 65.95 ± 3.85 years), healthy, had normal vision or vision that was corrected to normal, and had mental state test scores ≥24 points. The Capital University of Physical Education and Sports (CUPES) Ethics Committee (approval No. CUPES-2018-06-15-01) approved the study and all participants signed informed consent forms.

### Test tools

#### Yale physical activity scale

The YPAS is one of the scales that are commonly used to measure time spent in physical activity, energy consumption and related activity index [15]. It assesses the participant’s level of physical activity, recreational activities and domestic activities. The recall period is designated as a specific week in the month prior to the study. The target population includes healthy elderly people aged 60 and over.

The questionnaire consists of two parts. The first part lists 27 activities that are divided into three categories: household activities (including yard activities and care activities), exercise activities and entertainment activities. The respondents were asked to record the time (hours) spent on each activity in a week according to whether they performed the activity during an average week in the previous month. The total time is calculated by adding the time spent in all listed activities. Energy consumption (kcal/week) is calculated by multiplying the time spent in each activity by the corresponding intensity code.

The second part consists of nine questions designed to assess the participant’s level of participation in various types of physical activity, expressed as an index. The index of the five types of activities is calculated by multiplying the duration of each of the following activities by the specified weighting factor: (a) high-intensity activities; (b) leisure walking; (c) movement; (d) standing; (e) sitting. These indices are added to determine the sum index of the YPAS.

The time required to complete the YPAS is approximately 20 minutes. The questions that comprise the first part of the questionnaire are short and do not refer to difficult or controversial terms. The second part of the questionnaire includes questions about high-intensity physical activities and leisure walking; these need to be properly explained to the participants and the participants need to be given a clear definition of each activity.

#### Omegawave body function test system

The body function test system (Omegawave diagnostic system) can collect the nerve fatigue index and energy metabolism index of participants under laboratory conditions. The greatest advantage of this system is that it is easy to use and can be monitored in real time. The participants were tested in a supine position in a quiet environment. The test is divided into a “routine test” and a “complete test”. The routine test requires approximately 5 minutes to obtain an evaluation of the participant’s cardiac function and energy metabolism system. The complete test, which requires approximately 15 minutes, performs a functional evaluation of the participant’s central nervous system, cardiopulmonary, and liver transformation functions and his or her hormonal system.

The Omegawave diagnostic system is a new system that is designed to evaluate the state of physical function; it can measure VO2max and other indicators in elderly individuals. Research shows that the Omegawave diagnostic system has a high correlation with the results of traditional laboratory tests of function [18]. Traditional laboratory testing requires the elderly to do their best, and an Omegawave diagnostic system may be more suitable than other systems for testing the elderly.

The Omegawave diagnostic system was used in an environment in which the air humidity was 33% and the room temperature was 22 ± 1 °C. The test environment was quiet, and the lighting was appropriate. The participants did not consume any alcohol or drugs within the 24-hour period prior to the test. Before the test, the tester wetted the heart rate belt and assisted the participants in wearing the belt accurately. After putting on the heart rate belt, the participants were required to lie down for 1 min. The surface skin where the electrode sheet was to be applied was wiped with an alcohol-soaked cotton ball. The electrodes were applied to the thenar of the participant’s dominant hand and to the center of his forehead. After the instrument showed that the connection was successful, the machine was clicked to start the test. During the test, the participant was required to close his eyes, remain quiet, and refrain from moving or talking. The collection time was 4 minutes.

Relevant research shows that there is a significant correlation between aerobic capacity measured using the Omegawave diagnostic system and aerobic capacity measured using traditional test methods and that using the Omegawave system to test aerobic capacity is effective. The traditional aerobic capacity test requires participants to work as much as possible. The working characteristics of the Omegawave diagnostic system are more suitable for monitoring the aerobic capacity of the elderly.

### Experimental process

To ensure cultural equivalence between the simplified Chinese version of the YPAS and its original English version, a standardized translation procedure is used in this study, including six stages (initial translation, synthesis of the translations, back translation, test of the prefinal version and appraisal of the adaptation process) [19]. In the translation of the scale, it is assumed that the physical activity structure used in the YPAS is generally culturally suitable for individuals residing in communities in mainland China. In addition, the scale should be adapted to make it useful in cross-cultural research and comparison. Two scholars in the field of sports science analyzed the theoretical background of the questionnaire and considered the differences in culture and language in mainland China. The physical activity structure used in the YPAS is culturally suitable for communities in mainland China. Two independent bilingual scholars translated the questionnaire into Chinese and compared and retranslated the simplified Chinese version with respect to the original YPAS. Because courtyard activities are less often participated in by elderly groups in mainland China and gardening activities are more often participated in, “lawn cutting” and “cleaning trails” in the section on courtyard activities are replaced in the simplified Chinese version by “pruning bonsai” and “carrying flower pots”. In addition, because table tennis is very popular among the elderly in mainland China, “squash” is replaced by “table tennis” in the section on entertainment activities.

Each participant participated in the test twice. During the test, the basic information on the participant was first collected, and the test procedure was described to the participant in detail. The participants were familiar with the questionnaire and completed the scale to reflect their own actual situations. The participants had unlimited time to answer the questions on the YPAS. The entire filling process of completing the scale was guided by a trained master. After completing the scale, each participant wore the Omegawave diagnostic system and was tested by an experienced tester; the participant’s maximum oxygen consumption (VO2max) and energy metabolism system adaptation index (EMSAI) were recorded. One week later, the participant returned to the same place, completed the YPAS again, and participated in a second Omegawave test. The reliability of the YPAS was evaluated based on the consistency of the two measurements, and its validity was verified based on the relevant indicators of the Omegawave diagnostic system.

### Statistical methods

#### Test-retest reliability

The reliability of the YPAS was tested using a one-week interval retest procedure. SPSS (version 23.0; IBM Corp., Armonk, NY, United States) was used to process and analyze the data. The results were analyzed by descriptive statistics, intraclass correlation coefficient **(**ICC) and Pearson correlation analysis. The significance level was set at *p* < 0.05.

#### Criterion validity

The simultaneous validity was evaluated by comparing the results of the YPAS with those of the Omegawave diagnostic system. SPSS (version 23.0; IBM Corp.) was used to analyze the descriptive statistics and Pearson correlation of the data, and the significance level was set as *p* < 0.05.

## Results

### General description

A total of 21 participants completed all the tests. The average body mass index (BMI) of the 21 elderly participants was 24.09, a value that falls within the range of normal values. All participants had received a high school education or above, ensuring the quality of completion of the YPAS (Table 1).

**Table 1:** General description of participants

N	Factor	M	SD
21	Age (years)	65.95	3.85
	Height (m)	1.61	0.05
	Weight (kg)	62.67	7.66
	BMI	24.09	2.86

### Test-retest reliability

#### Physical activity time

Comparing the results of twice YPAS scales, it is found that the Pearson correlation coefficient and ICC on the total physical activity time of YPAS was high (*r* = 0.917, ICC = 0.897), indicating that the scale has good credibility in all participants. For the activity time subitem of YPAS, the Pearson correlation coefficient and ICC of housework activity time was the highest (*r* = 0.929, ICC = 0.923), while the reliability of exercise time (*r* = 0.892, ICC = 0.889) and entertainment time (*r* = 0.832, ICC = 0.803) was high (Table 2).

**Table 2:** YPAS scale - Reliability of pre-test and post-test of time

N	Factor	Pre-test	Post-test	Pearson	ICC
21	Housework activity time	33.29 ± 16.40	32.00 ± 14.53	0.929^*^	0.923^*^
	Exercise time	7.76 ± 4.60	7.43 ± 4.24	0.892^*^	0.889^*^
	Entertainment time	6.86 ± 4.56	8.05 ± 3.485	0.832^*^	0.803^*^
	Total time	47.91 ± 19.69	47.48 ± 15.95	0.917^*^	0.897^*^

*ICC* Intraclass correlation coefficient; *p < 0.05

**Fig. 1:**
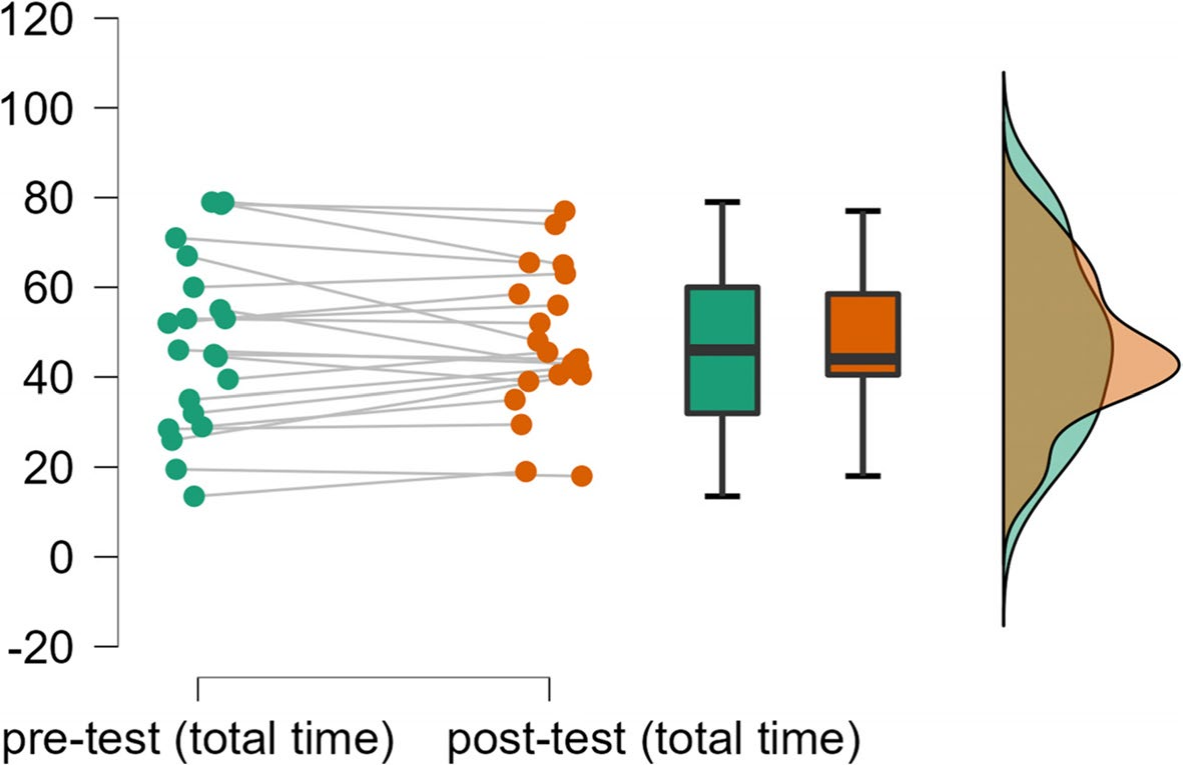
Reliability of pre-test and post-test of YPAS-physical activity total time

As shown in Fig. 1, the YPAS physical activity time of the two tests were highly coincident, and the degree of dispersion is low, indicating satisfactory reliability.

#### Calories consumed

Comparing the results of twice YPAS scales, it is found that the Pearson correlation coefficient and ICC on the total calories consumed was high (*r* = 0.958, ICC = 0.934), indicating that the scale has good reliability in all participants. For the exercise calories of YPAS, the Pearson correlation coefficient and ICC of housework activity time was the high (*r* = 0.956, ICC = 0.954), while the reliability of housework calories (*r* = 0.948, ICC = 0.933) and entertainment calories (*r* = 0.886, ICC = 0.872) was high (Table 3).

**Table 3:** YPAS scale - Reliability of pre-test and post-test of calories

N	Factor	Pre-test	Post-test	Pearson	ICC
21	Housework calories	6421.93 ± 3138.72	5919.76 ± 2626.987	0.948^*^	0.933^*^
	Exercise calories	2674.29 ± 1870.21	2560.17 ± 1758.34	0.956^*^	0.954^*^
	Entertainment calories	1550.71 ± 1240.23	1671.67 ± 1041.33	0.886^*^	0.872^*^
	Total calories	10,646.93 ± 4290.535	10,151.60 ± 3431.90	0.958^*^	0.934^*^

*ICC* Intraclass correlation coefficient; *p < 0.05

**Fig. 2:**
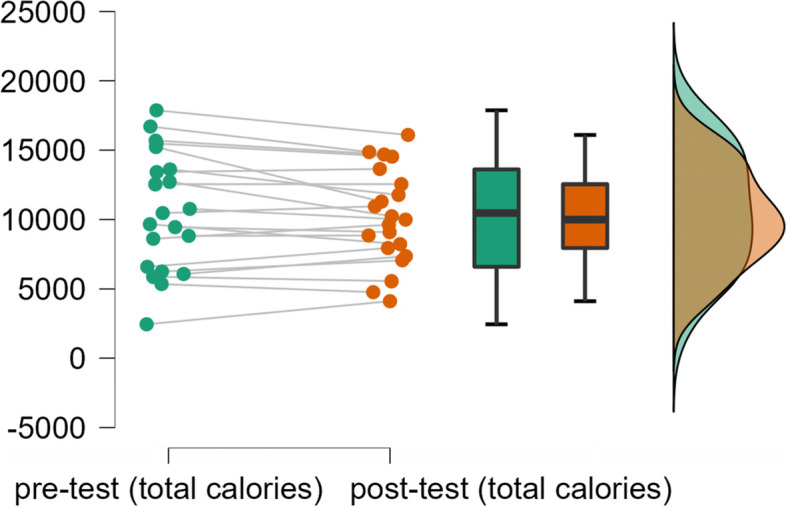
Reliability of pre-test and post-test of YPAS-total calories

As shown in Fig. 2, the YPAS-total calories of two tests were highly coincident, and the degree of dispersion is low, indicating satisfactory reliability.

#### Physical activity index

Comparing the results of twice YPAS scales, it is found that the Pearson correlation coefficient and ICC on the total index of YPAS was high (*r* = 0.930, ICC = 0.920), indicating that the scale has good credibility in all participants. For the high-intensity activity index of YPAS, the Pearson correlation coefficient and ICC of housework activity time was the highest (*r* = 0.942, ICC = 0.940), while the reliability of leisure walking index (*r* = 0.910, ICC = 0.902) and movement index (*r* = 0.587, ICC = 0.574), but the reliabilities of the standing index (*r* = 0.345, ICC = 0.342) and the sitting index (*r* = − 0.287, ICC = − 0.280) were low (Table 4).

**Table 4:** YPAS scale - Reliability of pre-test and post-test of physical activity index

N	Factor	Pre-time	Post-time	Pearson	ICC
21	High-intensity Activity index	8.33 ± 8.56	7.62 ± 8.00	0.942^**^	0.940^*^
	Leisure walking index	12.95 ± 6.19	11.81 ± 5.44	0.910^**^	0.902^*^
	Movement index	7.43 ± 2.94	6.90 ± 2.64	0.587^*^	0.574^*^
	Standing index	4.76 ± 1.73	4.38 ± 1.50	0.345	0.342
	Sitting index	1.33 ± 0.48	1.52 ± 0.60	−0.287	−0.280
	Total index	34.81 ± 12.98	32.24 ± 11.24	0.930^**^	0.920^*^

*ICC* Intraclass correlation coefficient; *p < 0.05; **p < 0.01

**Fig. 3:**
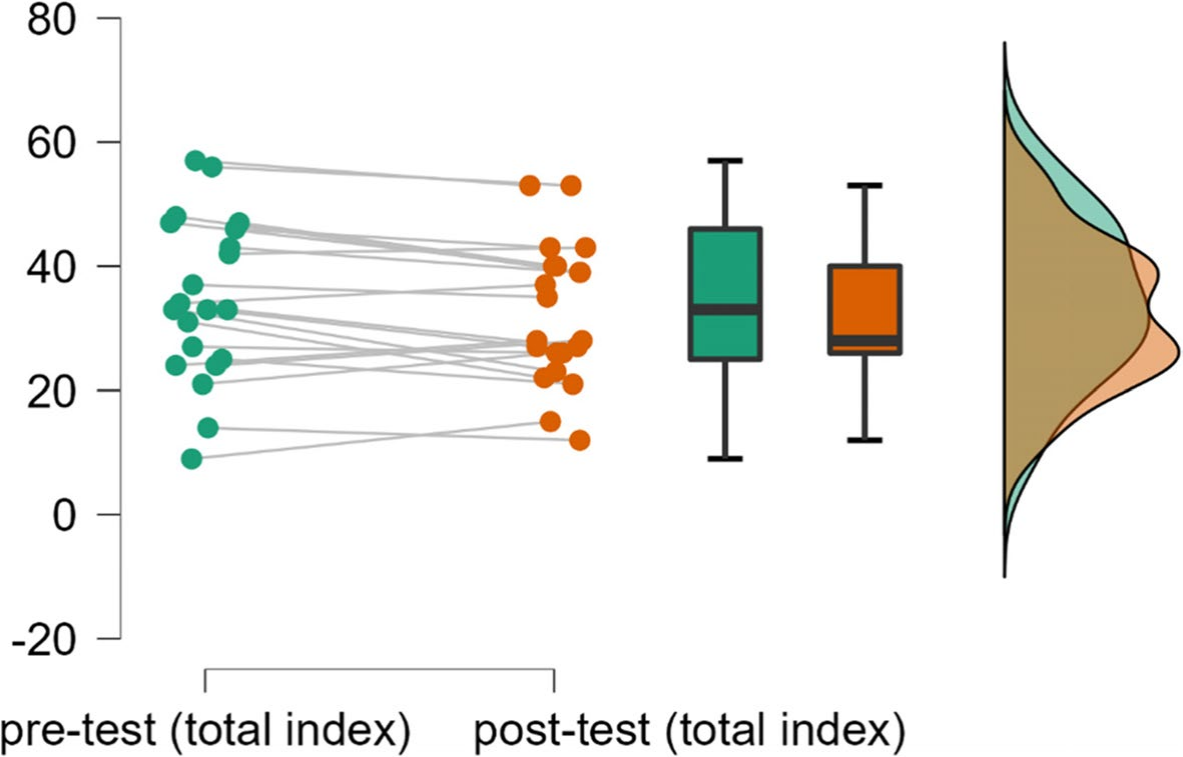
Reliability of pre-test and post-test of YPAS-total index

As shown in Fig. 3, the YPAS-total index of two tests were highly coincident, and the degree of dispersion is low, indicating satisfactory reliability.

### Criterion validity

#### Simultaneous validity

##### Physical activity time

The validity of the total time test in the second YPAS was verified using the energy metabolism system adaptation index of the Omegawave test. The results showed that there was a moderate correlation between them (*r* = 0.472), indicating that the YPAS had good test validity for participants (Table 5).

**Table 5:** Correlation between adaptation index of Omegawave energy metabolism system and total time and total calorie score of YPAS

Factor	*N* = 21	
	r	*p*
Total time	0.472^*^	0.031
Total calories	0.478^*^	0.028

*p < 0.05

##### Calories consumed

**Fig. 4:**
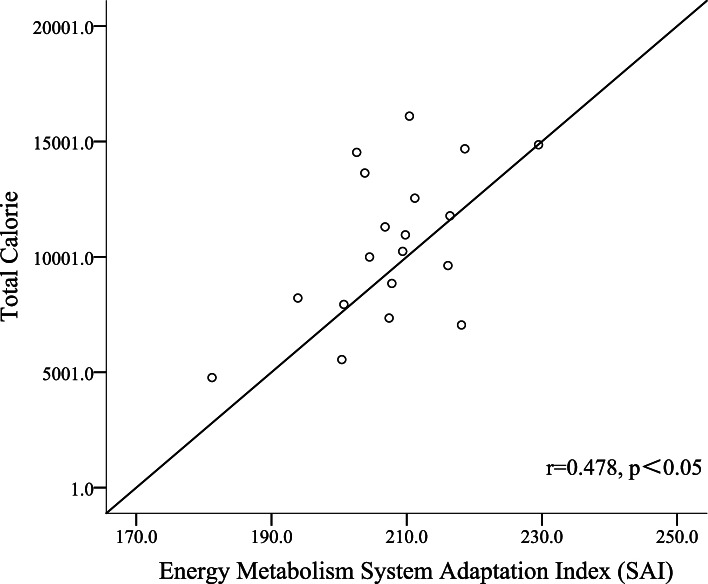
Validity of total calorie consumption of YPAS and Omegawave energy metabolism system adaptation index

On verifying the validity of the calorie consumption test in YPAS, it was found that there is a moderate correlation between the total calorie test value obtained using the YPAS and the adaptation index of the energy metabolism system obtained using the Omegawave test (*r* = 0.478), indicating that the YPAS has good test validity for these participants (Table 5, Fig. 4).

##### Physical activity index

The validity of the one-week YPAS was verified using the Omegawave diagnostic system. There was a high correlation between the total index score obtained on the YPAS and the maximum oxygen uptake obtained using the Omegawave system (*r* = 0.782), indicating that the YPAS had good test validity for these participants (Table 6).

**Table 6:** Correlation between maximum oxygen uptake of Omegawave and summation index of YPAS

Factor	*N* = 21	
	r	*p*
Summation index	0.782^**^	<0.01

**p < 0.01

## Discussion

The YPAS is a classic scale that is used to test the physical activity of elderly individuals worldwide, but most of the groups that have been used to test its reliability and validity are made up of elderly participants living in European and American countries. This study translated YPAS into simplified Chinese through a standardized translation process. In the translation of the scale, it is assumed that the physical activity structure used in the YPAS is generally culturally suitable for communities in mainland China. In addition, the scale was adapted to suit cross-cultural research and comparison. In this study, the reliability and validity of the YPAS were tested by comparing results obtained using the simplified Chinese version of the YPAS with results obtained using the Omegawave diagnostic system in a group of elderly women living in mainland China. The results show that the simplified Chinese version of the YPAS has high reliability and good validity.

When evaluating the physical activity of participants, methods such as behavioral observation, instrument tests and questionnaires are mainly used [20]. In research on physical activity, although the use of objective testing instruments to measure the level of physical activity yields highly accurate results, the cost of such testing is high, the programs involved are complex, and there are limitations that make it impossible to apply these instruments to large samples. The use of a standardized scale has the advantages of time savings and convenience in the investigation of physical activity in large sample groups.

The results of this study show that the YPAS has high reliability. After another measurement at an interval of 1 week, the correlations between the two measurement scores obtained by individuals in each dimension ranged from − 0.280 to 0.958, except for sitting index and standing index, showing good test-retest reliability. Relevant foreign studies have also found that the test-retest reliability of the YPAS is high [21]. In addition, the results of sitting index and standing index were unsatisfactory, possibly because its belong to relatively static movements, which are more difficult to be detected by the scale. However, the total index was reliable, and the YPAS mainly evaluated physical activity, therefore, the simplified Chinese version of the YPAS is a relatively stable tool for measuring physical activity in the elderly, except for sitting index and standing index, showing high cross-time consistency.

The results of this study show that the YPAS has high validity. Test validity is one of the key indicators of the application of a standardized scale. In this study, an objective testing instrument was used as the criterion to verify the physical activity scale. The validity of the verified scale is the key factor in determining whether the scale can be used in large sample investigations and research. The results of this study show that Omegawave can be used as an objective test instrument to evaluate the level of human energy metabolism. Using VO2max and the adaptation index of the energy metabolism system in the Omegawave diagnostic system as the criterion, it was verified that the simplified Chinese version of the YPAS has high validity.

There are deficiencies in this study. Although the sample size is insufficient, referring to the literature we found the effect size was acceptable, and this study provides useful information for helping the elderly know their physical activity and researchers in this field design their future work [22, 23]. Still, it is necessary to increase the sample size to further verify the reliability and validity of the results. In addition, the sample population of this study is insufficient. The participants in this study were mainly urban community residents, and the sample source was relatively singular. In future research, we should expand the scope of the sample for verification.

## Conclusion

The simplified Chinese version of the YPAS is suitable for measuring the level of physical activity and energy metabolism of elderly women in mainland China. It has high reliability and validity. It can be extended to large samples as a reliable test tool.

## Data Availability

All data generated and analyzed during this study are included in this published article. The raw data supporting the conclusions of this article will be made available by the authors, without undue reservation, to any qualified researcher. Yuan Yang should be contacted if someone wants to request the data from this study.

## References

[1] Hughes JP, McDowell MA, Brody DJ (2008). Leisure-time physical activity among US adults 60 or more years of age: results from NHANES 1999-2004. J Phys Act Health.

[2] Groot C, Hooghiemstra AM, Raijmakers PG (2016). The effect of physical activity on cognitive function in patients with dementia: a meta-analysis of randomized control trials. Ageing Res Rev.

[3] Resnick B, King A, Riebe D (2008). Measuring physical activity in older adults: use of the community health activities model program for seniors physical activity questionnaire and the Yale Physical Activity Survey in three behavior change consortium studies. West J Nurs Res.

[4] Król-Zielinska M, Ciekot M (2015). Assessing physical activity in the elderly: a comparative study of most popular questionnaires. Trends Sport Sci.

[5] Forsen L, Loland NW, Vuillemin A (2010). Self-administered physical activity questionnaires for the elderly: a systematic review of measurement properties. Sports Med.

[6] Jorstad-Stein EC, Hauer K, Becker C (2005). Suitability of physical activity questionnaires for older adults in fall prevention trials: a systematic review. J Aging Phys Act.

[7] Shephard R (2003). Limits to the measurements of habitual physical activity by questionnaires. Br J Sports Med.

[8] Mro A, Ips A, Vdmk B (2021). Covid-19 and the impact on the physical activity level of elderly people: a systematic review. Exp Gerontol.

[9] Król-Zielińska M, Zieliński J, Kantanista A (2019). Polish adaptation of the Yale Physical Activity Survey: measurement properties. Int J Public Health.

[10] Dishman RK, Heath GW, Washburn R. Physical activity epidemiology. Champaign: Human Kinetics; 2004.

[11] Martin V, Ayan C, Molina AJ (2012). Correlation between the Yale Physical Activity Survey (YPAS) and a submaximal performance-based test: a study in a population of elderly Spanish women. Arch Gerontol Geriatr.

[12] Semanik P, Lee J, Manheim L (2011). Relationship between accelerometer-based measures of physical activity and the Yale Physical Activity Survey in adults with arthritis. Arthritis Care Res.

[13] Kolbe-Alexander TL, Lambert EV, Harkins JB (2006). Comparison of two methods of measuring physical activity in South African older adults. J Aging Phys Act.

[14] Gennuso KP, Matthews CE, Colbert LH (2015). Reliability and validity of two self-report measures to assess sedentary behavior in older adults. J Phys Act Health.

[15] Di Pietro L, Caspersen C, Ostfeld A (1993). A survey assessing physical activity among older adults. Med Sci Sports Exerc.

[16] Young DR, Jee SH, Appel LJ (2001). A comparison of the Yale Physical Activity Survey with other physical activity measures. Med Sci Sports Exerc.

[17] The Lancet Public Health (2019). Time to tackle the physical activity gender gap. Lancet Public Health.

[18] Naranjo-Orellana J, Ruso-Lvarez JF, Rojo-Lvarez JL (2020). Correction: comparison of Omegawave device and an ambulatory ECG for RR interval measurement at rest. Int J Sports Med.

[19] Beaton DE, Bombardier C, Guillemin F (2000). Guidelines for the process of cross-cultural adaptation of self-report measures. Spine (Phila Pa 1976).

[20] Paffenbarger RJ, Blair SN, Lee IM (1993). Measurement of physical activity to assess health effects in free-living populations. Med Sci Sports Exerc.

[21] Pennathur A, Magham R, Contreras LR (2004). Test-retest reliability of Yale Physical Activity Survey among older Mexican American adults: a pilot investigation. Exp Aging Res.

[22] Kruskall LJ, Campbell WW, Evans WJ (2004). The Yale Physical Activity Survey for older adults: predictions in the energy expenditure due to physical activity. J Am Diet Assoc.

[23] Moore DS, Ellis R, Allen PD (2008). Construct validation of physical activity surveys in culturally diverse older adults. Res Q Exerc Sport.

